# *Anabaena/Dolichospermum* as the source of lethal microcystin levels responsible for a large cattle toxicosis event

**DOI:** 10.1016/j.toxcx.2018.100003

**Published:** 2018-12-10

**Authors:** Theo W. Dreher, Lindsay P. Collart, Ryan S. Mueller, Kimberly H. Halsey, Robert J. Bildfell, Peter Schreder, Arya Sobhakumari, Rodney Ferry

**Affiliations:** a Department of Microbiology, Oregon State University, Corvallis, OR 97331, USA; b Center for Genome Research and Biocomputing, Oregon State University, Corvallis, OR 97331, USA; c Department of Biomedical Sciences, Oregon State University, Corvallis, OR 97331, USA; d Oregon State University Extension Service, Lakeview, OR 97630, USA; e California Animal Health and Food Safety Laboratory, Davis, CA 96617, USA; f Lakeview Animal Hospital, Lakeview, OR 97630, USA

**Keywords:** Anabaena sp. JUN03, Cyanobacteria, Cyanotoxin, Cattle mortalities, Microcystin, Mcy genes

## Abstract

Thirty-two 14-month old steers died during a period of four days (19–23 June 2017) after drinking from Junipers Reservoir (southeastern Oregon, USA) during a cyanobacterial bloom. Clinical and histopathological findings were consistent with acute liver disease, and microcystin-LR was present at 3000 μg/L in a reservoir water sample and at 7100 μg/L in the rumen contents of one of the mortalities. Serum biochemistry and histological examination indicated severe liver damage consistent with microcystin toxicosis. Microscopic observation of reservoir water samples, limited to frozen or poorly stored and partially degraded samples, indicated the presence of abundant *Anabaena/Dolichospermum,* but the presence of other toxic cyanobacteria such as *Microcystis* could not be excluded. Metagenomic analysis showed the presence in these samples of a single cyanobacterium whose *cpcBA, rpoB* and *rbcL* genes indicated membership in the *Anabaena/Dolichospermum* genus. The sequence of a complete *mcy* gene cluster with homology to previously identified *Anabaena mcy* genes was recovered. These results emphasize the capacity for *Anabaena/Dolichospermum* blooms to produce lethal levels of microcystin, posing a danger to public health and livestock. Further, our findings indicate that such occurrences can occur outside the far-northern latitudes in which microcystin-producing *Anabaena* have typically been found.

## Highlights

• Death of 32 cattle emphasizes the danger of livestock poisoning by cyanobacterial toxins.
• Deaths resulted from microcystin-LR toxicosis.
• The producing organism was genetically identified as a member of the *Anabaena/Dolichospermum* genus.
• *Anabaena/Dolichospermum* blooms can be as potent a source of microcystins as is common for *Microcystis* or *Planktothrix*.

## Graphical abstract

**Figure:**
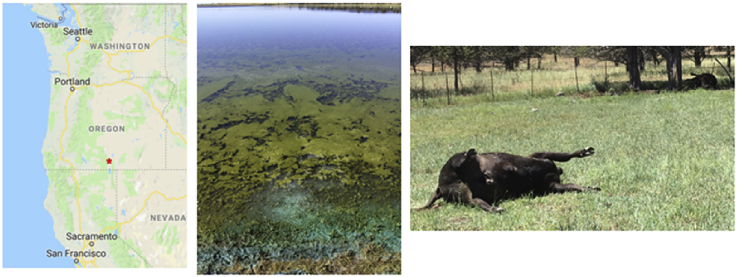

## 1 Introduction

Toxins originating from freshwater cyanobacterial blooms represent a widespread risk to humans, pets and livestock. Such blooms threaten the safety of recreational and drinking waters for human use (Backer et al., 2015, Chorus and Bartram, 1999), and they are recognized as a common, though under-documented, cause of deaths of pets, particularly dogs (Backer et al., 2013). Cyanobacterial blooms are also of concern for livestock in both farm and rangeland settings, with toxicosis episodes being reported in many parts of the world (Briand et al., 2003) and farm extension bulletins commonly raising general awareness of the threats of cyanotoxicosis. Microcystins are often involved (Briand et al., 2003, Mez et al., 1997, Odriozola et al., 1984), but nodularin, anatoxin-a, cylindrospermopsin and saxitoxin have also contributed to serious livestock mortalities (Falconer, 2001, Thomas et al., 1998).

The microcystins form a group of over 100 variants of hepatotoxic seven-membered cyclic peptides that are synthesized by a c. 50-kbp *mcy* gene cluster comprised of nonribosomal peptide synthetase (NRPS) and polyketide synthase (PKS) genes (Dittmann et al., 2012). The microcystins are recognized in all parts of the world as widespread and common cyanotoxins, presenting a serious threat to public health (Backer et al., 2015, Chorus, 2012, Chorus and Bartram, 1999); the LD_50_ of microcystin -LR in mice is 60 μg/kg (Slatkin et al., 1983). Microcystins have been detected in one-third to one-half of lakes sampled in the USA (Graham et al., 2010, Loftin et al., 2016) and in 85% of lakes sampled in Poland (Kobos et al., 2013). While a range of genera contain members capable of producing microcystin, *Microcystis* and *Planktothrix* are generally considered to be the most potent sources, and are often associated with high levels of the toxin (Chorus, 2012, Chorus and Bartram, 1999, Fastner et al., 1999). Studies in a number of U.S. lakes (Hotto et al., 2008, Rinta-Kanto et al., 2009, Rinta-Kanto and Wilhelm, 2006), including the Pacific Northwest (Bozarth et al., 2010, Jacoby and Kann, 2007), and in Europe (Kobos et al., 2013, Lahti et al., 2001, Vaitomaa et al., 2003), have also shown that the occurrences of microcystins or *mcy* genes are most commonly associated with these two genera.

The filamentous, nitrogen fixing genus *Anabaena/Dolichospermum* is also known to contain members capable of producing microcystins (Li et al., 2016). Such isolates have been reported mainly from northern latitudes in Europe (Kobos et al., 2013, Rantala et al., 2006, Rapala et al., 1997, Sivonen et al., 1992) and Canada (Harada et al., 1991, Krishnamurthy et al., 1986). *Anabaena/Dolichospermum* has thus been considered as a potential microcystin producer in risk assessments (Chorus and Bartram, 1999), but observations linking high levels of microcystin to *Anabaena/Dolichospermum* in lower latitudes, including the U.S., have been lacking, such that this genus is often not under high suspicion of being responsible for serious microcystin toxicosis events. Here, we document an event from southern Oregon (USA) in which 32 steers died after ingesting lake water containing an *Anabaena/Dolichospermum* bloom, and present evidence that the high level of microcystin-LR produced by *Anabaena* sp. JUN03 was the cause of death. Our observations show that *Anabaena/Dolichospermum* can produce potent levels of microcystin, and that such toxicosis events are not limited to the high northern latitudes.

## 2 Material and methods

### 2.1 Sample collection and field diagnosis

The cattle deaths occurred at Junipers Reservoir (∼30 ha) near Lakeview, Oregon, GPS coordinates 42.193647, −120.527233, beginning on 19 June 2017. The animals were 14 month-old Angus cross steers of about 340 kg body weight. Field necropsies were conducted on 19 June and 21 June, when blood and organ samples (heart, lung, kidney, liver, spleen, gastrointestinal tract and lymph nodes) were collected for submission for blood chemistry and histopathological analysis (Oregon Veterinary Diagnostic Laboratory, Oregon State University).

On 20 June 2017, samples were taken from three farm drinking water sources, including Junipers Reservoir (sample JUN03), and from the rumen of a recently deceased steer. The samples were frozen and sent for microcystin analysis (California Animal Health and Food Safety Laboratory, University of California-Davis). Aliquots of these samples were sent (frozen) to Oregon State University for genetic analysis. Another sample (JUN01) containing scum was taken from Junipers Reservoir on 21 June 2017 and mailed on ice to Oregon State University for genetic analysis; this sample was detained in transit for four days, exposing it to elevated temperatures.

### 2.2 Histopathological analysis

The above-mentioned tissues from two steers that had been fixed in 10% neutral buffered formalin were processed and embedded in paraffin. Six μm-thick tissue sections were cut and stained with hematoxylin and eosin for examination by light microscopy.

### 2.3 Toxin analysis

Water and rumen samples were analyzed for microcystins -LA, -LR, -RR and -YR by LC-MS/MS (Sciex 4000 QTrap triple quadrupole/ion trap mass spectrometer). Samples were sonicated, followed by five freeze/thaw cycles to release toxins from cells. After filtration, toxins were concentrated using solid phase extraction columns, evaporated to dryness and redissolved in methanol/water. Data were acquired from the mass spectrometer in the positive ion electrospray mode, using the following TurboIonSpray source conditions: temperature 500 °C, curtain gas set to 30 (arbitrary units), GS1 and GS2 to 50 and 50 respectively, CAD gas pressure high, ion spray voltage set to 5500. The precursor and product ions monitored are indicated in Suppl. Table S1.

### 2.4 Phytoplankton analysis by microscopy

Junipers Reservoir water samples JUN01 and JUN03 were examined by microscopy (bright field and phase contrast) at magnifications of 100× to 400×. Cyanobacterial identification was made with reference to previous descriptions (Komárek and Mareš, 2012, Wehr et al., 2002).

### 2.5 Genetic analyses

DNA was prepared from sample JUN03 (previously frozen scum-containing water sample from Junipers Reservoir) by cell lysis with lysozyme, proteinase K and sodium dodecylsulfate followed by phenol/chloroform extraction (Bozarth et al., 2010) and purification using a DNeasy PowerBiofilm DNA isolation kit (Qiagen). Cellular material from sample JUN01 (never-frozen Junipers Reservoir scum-containing water sample) was collected by filtration on 1.2 μm pore size glass fiber filters (VWR), and DNA was then extracted and purified from the sample using the DNeasy PowerBiofilm kit, with bead beating for cell lysis. DNA was extracted from a sample of rumen contents using a DNeasy Blood and Tissue DNA isolation kit (Qiagen).

DNA extracted from JUN01 and JUN03 samples was used for metagenome sequencing at the Center for Genome Research and Biocomputing at Oregon State University. JUN03 DNA was used to construct a DNA library with ∼800 bp insert size using a Nextera XT kit. The JUN01 DNA sequencing library was produced via TruSeq PCR-free methodology, beginning with 2 μg DNA and targeting an insert size of ∼470 bp. Libraries were sequenced on an Illumina HiSeq 3000 instrument generating paired-end 2 × 151 nucleotide reads.

Sequencing reads were quality screened as previously described (Otten et al., 2016) and mapped to reference sequences (Suppl. Tables S3 and S4) using the default settings of BowTie2 (Langmead and Salzberg, 2012). Further analyses using Geneious DNA analysis software included consensus contig assembly covering the microcystin biosynthesis genes from mapped reads using the SPades 3.10.0 assembler (Nurk et al., 2013) and detection of single nucleotide polymorphisms against generated consensus sequences.

To verify uncertainties in the assembled DNA sequences in regions of high homology in the *mcyB* and *mcyC* genes, polymerase chain reaction (PCR) fragments were amplified from JUN03 DNA and submitted for Sanger sequencing using an ABI 3730 capillary instrument by the Center for Genome Research and Biocomputing at Oregon State University. Amplification used the primers McyBir-F and McyBir-R and annealing temperatures described in Suppl. Table S5.

PCR was also used to verify the presence of microcystin-producing *Anabaena/Dolichospermum* in the rumen sample (primers identified in Suppl. Table S5).

## 3 Results and discussion

### 3.1 Cattle deaths

Thirty-two cattle died over a 4-day period near Lakeview, OR, beginning on 19 June 2017, a hot day of 36 °C. A northerly wind had concentrated a cyanobacterial bloom to form scum at the southern end of Junipers Reservoir, where a herd of 207 14-month-old steers had access to the lake via a short stretch of shoreline (P. Schreder, R. Ferry, on-site observations). Blue-green stains were evident on the legs and bellies of the cattle, indicating they had waded into the reservoir. The most rapidly dying animals (19 June) showed signs of excitation, head tremors and staggering gait that progressed very rapidly to tetany and death, with or without convulsions. Most acute signs appeared to be neurological.

Surviving cattle were moved to another water source on 19 June. Daily deaths were recorded as six on 19 June, 11 on 20 June, 11 on 21 June, one on 22 June, and an additional three by 23 June, totaling 32. Two recently deceased animals were necropsied in the field on 19 June and 21 June, revealing pale livers (and spleen in one animal) as the only abnormalities. Although the symptoms were mainly neurological in nature, initially suggesting the possibility of neurotoxin poisoning (Briand et al., 2003), the extended period of mortality (over a five day period) and the abnormal livers revealed by the field autopsies pointed to the action of a hepatotoxin.

Documented symptoms of acute microcystin poisoning are rather non-specific in nature, including paleness due to reduced blood supply to the periphery, and muscle weakness and recumbency observed as common symptoms across multiple mammalian species, including cattle (Elleman et al., 1978, Galey et al., 1987, Konst et al., 1965). Diarrhea or vomiting have been observed in some cases (Briand et al., 2003, Galey et al., 1987), but not in others. Indeed, neurological symptoms such as muscular twitching, convulsive spasms, paralysis or muscle fibrillations are more commonly cited in experiments involving administration of purified microcystin toxin to mammals (Elleman et al., 1978, Falconer et al., 1981, Konst et al., 1965, Slatkin et al., 1983), including cattle (Konst et al., 1965). The symptoms observed in the steers described in this report fall within the range of microcystin toxicosis symptoms reported in the literature.

### 3.2 Presence of high levels of microcystin-LR

The most likely toxin candidate was microcystin, which has been present at high levels in recent years in the nearby Klamath basin (Upper Klamath Lake and downstream reservoirs on the Klamath River) about 70 km to the west (KBMP, 2018, Otten et al., 2015). Consequently, quantitative analyses for four microcystin congeners (-LA, -LR, -RR and -YR) were conducted on the rumen contents of one recently deceased steer and on samples (20 June) of the three water bodies available to the animals: Junipers Reservoir and two small ponds. A single congener, microcystin-LR, was detected at concentrations of 3000 μg/L in the JUN03 Junipers Reservoir sample and 7100 μg/L in the rumen sample (Table 1). Microcystin-LR is the most common, and one of the most toxic, microcystin congeners, and the WHO-recommended guideline value of 1 μg/L is commonly used for assessing drinking water safety (WHO, 2003).

**Table 1:** Microcystin concentrations determined by LC-MS. ND, not detected. In addition to Junipers Reservoir, House pond and Small pond were water sources available to the steers.

	Microcystin concentration (μg/L)			
	Microcystin-LA	Microcystin-LR	Microcystin-RR	Microcystin-YR
Rumen contents	ND	7100	ND	ND
Junipers Reservoir (JUN03)	ND	3000	Trace (<10)	ND
House pond	ND	ND	ND	ND
Small pond	ND	ND	ND	ND

### 3.3 Serum biochemistry and histopathology consistent with acute hepatotoxin exposure

A biochemical profile performed on serum collected (19 June) from a steer just prior to death on the first day of the toxicosis event revealed several values strongly indicative of hepatic damage (Table 2). The full analysis is available in Suppl. Table S2.

**Table 2:** Highly elevated markers for liver damage in serum of acutely poisoned steer.

	Concentration in steer serum	Normal concentration range
Aspartate aminotransferase	>5000 U/L	51–114 U/L
Sorbitol dehydrogenase	>170 U/L	0–50 U/L
Gamma glutamyl transferase	100 U/L	1–31 U/L
Alkaline phosphatase	1102 U/L	30–190 U/L
Total bilirubin	2.8 mg/dL	0.1–0.5 mg/dL

**Fig. 1:**
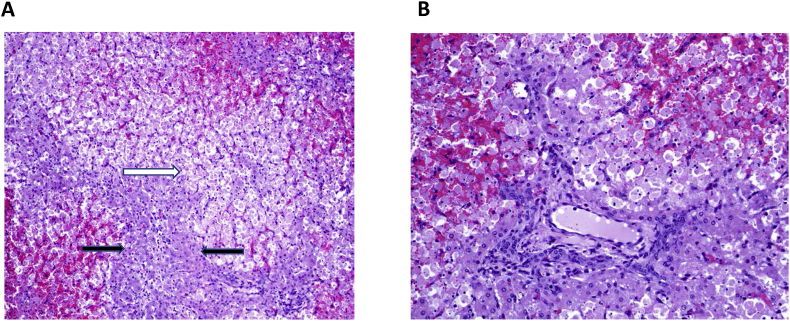
Severe hepatic lesions in steer suffering acute toxicosis and death, 19 June 2017. A, B: Hematoxylin/eosin stained hepatic sections showing acute necrosis, post-necrotic collapse and severe hepatic vacuolation with hemorrhage. **A.** Note extensive necrosis of tissue to right of white arrow. Some periportal hepatocytes have been spared (located between black arrows); 100X. **B.** Hemorrhage evident between necrotic, dissociated hepatocytes above the portal area. Some intact hepatocytes persist below the portal area. Cholangiolar cells are mildly hyperplastic. 200X.

Histological examination of tissues from the same animal revealed massive hepatic necrosis, obliterating some lobules and sparing only small numbers of periportal hepatocytes in others (Fig. 1A and B). Necrotic areas tended to be congested due to pooling of erythrocytes between the dissociated hepatocytes in hepatocellular cords. Cholangiolar cells were prominent and a few neutrophils were visible in some of the periportal connective tissues, but overall there was minimal response to this extensive damage, suggesting a peracute to acute process. Some other tissues had minor changes interpreted as incidental, including a mild increase in proprial neutrophils and eosinophils in the small intestine. The section of heart featured focally extensive subendocardial hemorrhage and the myocardium contained a few sarcocysts. Lung, spleen, kidney, and lymph node lacked significant lesions.

The hepatic tissue from a second steer whose death was delayed (21 June) was autolytic and more difficult to assess microscopically. Hepatocytes in centrilobular areas were poorly defined with only a few convincingly necrotic cells seen amid a collapse of tissue in this zone. Midzonal and periportal hepatocytes contained a few sharply demarcated cytoplasmic vacuoles (interpreted as lipid). The heart of this animal also contained a few sarcocysts, while only autolytic change was identified in lung, spleen and kidney.

These findings are consistent with microcystin toxicosis. Administration of lethal microcystin doses to a range of mammals, including cattle, has resulted in pathology heavily focused on the liver, with vacuolation, hepatocyte necrosis and hemorrhage the dominant symptoms (Elleman et al., 1978, Falconer et al., 1981, Galey et al., 1987, Konst et al., 1965). Serum biochemical changes and histopathologic findings in the first steer are typical of acute cyanobacterial toxicosis (Galey et al., 1987), while subacute cases (second steer) are often characterized by less extensive necrosis but increased fatty change (Cullen and Stalker, 2015).

### 3.4 Microscopy evidence for abundant Nostocales in Junipers Reservoir

**Fig. 2:**
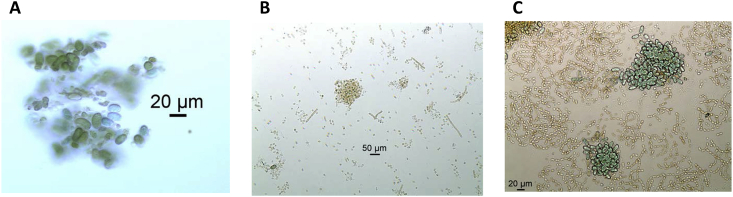
Microscopy of scum-containing water samples from Junipers Reservoir. **A.** Sample JUN03: *Nostocales* akinetes were the only intact cells evident after freezing and thawing. 200×, unstained. **B.** Sample JUN01: collapsed colonies or filament tangles with intact akinetes (large ovals) and heterocysts (spherical dark green cells) indicative of *Anabaena/Dolichospermum*, together with unidentified individual cells from dispersed colonies or filaments and occasional straight filaments. 100×. **C.** Sample JUN01: Clumps of akinetes surrounded by relatively intact *Anabaena/Dolichospermum* filaments. The morphology is similar to that of the *Anabaena lemmermannii/flos aquae/mendotae* cluster of species. 200×.

Both samples of scum-containing water obtained from Junipers Reservoir provided limited opportunity for analysis by microscopy. For sample JUN03 (20 June), this was the result of freeze/thaw cycles causing actual or potential lysis of many cells, although the sample was fresh in appearance due to continual storage at either 4 °C or frozen. Abundant green oblong cells (7-11 × 15–25 μm in size) present in clumps were the only intact cells observed (Fig. 2A). These were interpreted as akinetes (spore-like cells) diagnostic of the filamentous *Nostocales* cyanobacteria (Komárek and Mareš, 2012, Wehr et al., 2002).

Sample JUN01 (21 June) spent several days at elevated temperature during shipment and was brown and partially putrefied. Nevertheless, a few filaments and many intact, solitary, putative cyanobacterial cells were evident, though most of these were unidentifiable individual cells presumably released from colonies or filaments (Fig. 2B). Also present were clumps containing intact akinetes and heterocysts diagnostic of *Anabaena/Dolichospermum* (Fig. 2B and C; Suppl. Fig. 1). These differentiated cells appeared to be embedded in clumps of collapsed vegetative cells. In some cases, intact filaments containing vegetative cells were evident (Fig. 2C).

While *Anabaena/Dolichospermum* was abundant, the possible presence of other cyanobacteria that could be toxin producers was considered. The individual cells shown in Fig. 2B could have originated from *Microcystis* colonies, although no colonies were evident. Occasional filament fragments with rectangular cells suggestive of *Aphanizomenon flos-aquae* (distinct from the rounded *Anabaena/Dolichospermum* cells) were observed (Fig. 2B; Suppl. Fig. 1A).

No definitive identification of cyanobacterial cells could be made in the rumen sample.

### 3.5 Metagenome analysis shows presence of microcystin genes derived from a single *Anabaena*/*Dolichospermum* strain

Metagenome libraries were created and sequenced for the JUN01 and JUN03 Junipers Reservoir water samples with the objective of identifying the sequence and origin of cyanotoxin biosynthetic genes as well as taxonomic diversity of cyanobacteria present in the sample. The JUN01 sample was concentrated by filtration onto 1.2 μm glass fiber filters, which also removes many heterotrophic bacterial cells while retaining the larger cyanobacterial cells, before DNA extraction. The entire JUN03 sample was used for DNA extraction because its exposure to freezing and thawing could have released the DNA of some cells. Because cyanobacterial cells could not be enriched, one-third of a lane on an Illumina HiSeq 3000 instrument was used (see Materials and Methods) to ensure the acquisition of enough sequencing reads to detect minor population members.

**Fig. 3:**
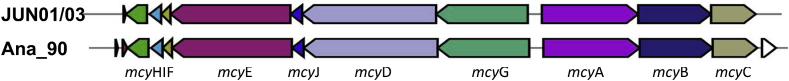
The 55-kbp *mcy* gene cluster derived from JUN01 and JUN03 metagenomes has the same gene arrangement as the *mcy* cluster from *Anabaena* sp. 90. Overall nucleotide identity = 98.8%.

The JUN01 and JUN03 metagenome libraries consisted of 24,944,470 and 133,296,655 paired DNA reads, respectively, representing 3760 and 20,128 million nucleotides sequenced. The sequencing reads from each library were recruited to the 5.31 Mbp genome of *Anabaena* sp. 90, which initial comparisons indicated was closely related to the principal cyanobacterial sequences present. The *Anabaena* sp. 90 genome includes a 56 kbp region containing the *mcyHIFEJDGABC* genes for microcystin synthesis (Wang et al., 2012) (Fig. 3). Reads were also recruited to a custom file containing sequences of cyanotoxin genes, including microcystin biosynthetic genes from *Anabaena*, *Microcystis* and *Planktothrix,* and genes for anatoxin-a, cylindrospermopsin, nodularin and saxitoxin biosynthesis (Suppl. Table S3). The *Anabaena* sp. 90 *mcy* gene cluster was the only one of these sequences that recruited reads to more than one or two genes or domains from the query sequence.

Both JUN01 and JUN03 metagenomes contained *mcyHIFEJDGABC* gene clusters identical in organization to that of *Anabaena* sp. 90 (Fig. 3). The average read depths across the *mcy* genes were 447× and 1493× for JUN01 and JUN03, respectively (Table 3). Identical *mcy* consensus sequences were derived from the two metagenomes, with no nucleotide polymorphisms above 5% present. Because 1.6 kbp of the *mcyB* and *mcyC* genes are highly homologous, initial read mapping left some sequence uncertainties in these genes. Sequencing of PCR products amplified with the McyBir PCR primers (Suppl. Table S5) revealed the presence of a 72 bp insertion in *mcyC* relative to the homologous part of *mcyB* (Suppl. Fig. S2) and relative to *Anabaena* sp. 90 *mcyC* (Suppl. Fig. S3). Read mapping (both JUN01 and JUN03) to a corrected reference genome bearing this insertion resolved all consensus sequence ambiguities; the absence of nucleotide polymorphisms at >5% was verified for the corrected *mcyBC* consensus sequences. The nucleotide sequence of the 54,926 bp *mcyHIFEJDGABC* gene cluster has been deposited in GenBank under the organism name *Anabaena* sp. JUN03 (Accession number MH663498).

**Table 3:** Genes identified by read mapping from metagenomes JUN01 and JUN03 to homologous genes from *Anabaena* sp. 90.

Gene(s)	JUN01 Av. Read depth	JUN03 Av. Read depth	Nucleotide identity to Anabaena sp. 90 homologs (%)	GenBank accession No.^c^
*mcyHIFEJDGABC*	447	1493	98.92	MH663498
Other toxin genes^a^	None detected	None detected		
*cpcBA*^b^	350	1161	99.54	MH663497
*rbcL*	332	1378	99.44	MH663499
*rpoB*	378	1166	98.98	MH663500

a Biosynthetic genes for the following toxins: nodularin, anatoxin-a, cylindrospermopsin, saxitoxin (see Suppl. Table S3).

b *cpcBA* includes *cpcB* and *cpcA* coding regions and intergenic region.

c Sequences appear in GenBank under the organism name *Anabaena* sp. JUN03.

AntiSMASH v4.1.0 (Blin et al., 2013) predicted similar products catalyzed by the JUN01/03 and *Anabaena* sp. 90 Mcy proteins, with leucine predicted as the amino acid inserted at the two variable sites in microcystin, to produce microcystin-LL. The same prediction was made by SEQL-NRPS (Knudsen et al., 2015). These amino acids are selected by the N-terminal adenylation domain of McyB and the sole adenylation domain of McyC (Rouhiainen et al., 2004), which are closely homologous in both *Anabaena* sp. 90 and JUN01/03 despite the 24-amino acid insertion in the JUN01/03 *mcyC* adenylation domain. *Anabaena* sp. 90 has been shown to produce 57% microcystin-LR, 15% [D-Asp^3^]microcystin-LR and 28% microcystin-RR; no microcystin-LL has been reported (Sivonen et al., 1992). Bioinformatic prediction of *Anabaena* sp. 90 NRPS specificity is clearly imperfect; this is also true for the JUN01/03 NRPS, which we have shown to produce microcystin-LR (Table 1), although it cannot at present be excluded that some microcystin-LL is produced.

Two additional read mapping analyses were conducted to look for evidence of other cyanobacteria in the JUN01 and JUN03 samples. First, the reads were mapped to a suite of cyanobacterial genomes, including several *Nostocales*, *Microcystis* and *Synechococcus*, representing cyanobacteria that might be expected to be present in an Oregon lake sample (Suppl. Table S4). Reads were most efficiently mapped to the genome of *Anabaena* sp. 90 and its close relatives. Reads mapping to other genomes covered only fragments of the genomes, reflecting the presence of widely conserved genes. Second, reads were mapped to a reference file containing the *cpcBA*, *rbcL* and *rpoB* genes from *Anabaena* sp. 90, *Nostoc* PCC 7120 and *Cyanobium gracile* PCC 6307. These genes have been used in cyanobacterial phylogenetic analyses (Li et al., 2016, Rajaniemi et al., 2005) and this choice of organisms broadly represents the *Nostocales* and the common freshwater *Synechococcus* group of picocyanobacteria. Reads mapped to the *cpcBA*, *rbcL* and *rpoB* genes from *Anabaena* sp. 90 at depths similar to that for the *mcy* genes (Table 3), generating consensus sequences for which no polymorphisms were present at a 5% cutoff. The small number of reads mapped to the *Nostoc* and *Cyanobium* genes did not cover the entire query sequence and most of these reads were also homologous to *Anabaena* sp. 90 sequences. Both these approaches found no evidence for more than one cyanobacterium present in the Junipers Reservoir samples.

**Fig. 4:**
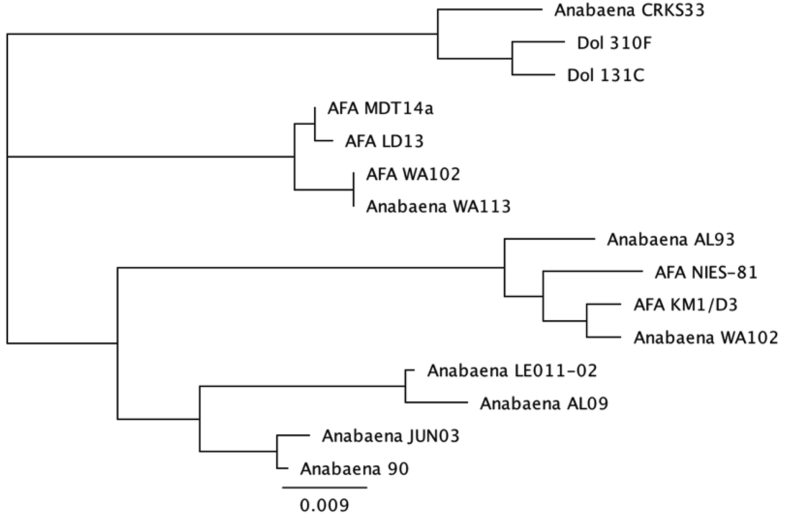
Phylogenetic relationship between the *cpcBA* gene of *Anabaena* sp. JUN03 and other *Nostocales* members of the ADA clade (Driscoll et al., 2018). The neighbor-joining phylogenetic tree was constructed after alignment of full-length *cpcBA* gene sequences. Dol, *Dolichospermum circinale*; AFA, *Aphanizomenon flos-aquae*.

Phylogenetic analysis of the JUN03 *cpcBA* gene sequence (Fig. 4) indicated membership of the recently recognized ADA clade of bloom-forming *Nostocales* (Driscoll et al., 2018), with *Anabaena* sp. 90 as the closest member. Similar relationships were observed for the *rbcL* and *rpoB* genes (not shown).

### 3.6 Rumen contents contain toxic *Anabaena*/*Dolichospermum*

DNA was extracted from a sample obtained from the rumen of a deceased steer and PCR amplifications were conducted to detect cyanobacterial genes. Primers specific for the *mcyB* and *mcyC* genes amplified the same doublet observed with the JUN01 and JUN03 samples (Suppl. Fig. S2). Primers specific for *Anabaena/Dolichospermum* 16S rDNA genes amplified the same size product from rumen, JUN01 and JUN03 samples (Suppl. Fig. S2). These observations indicate the presence of genomic DNA representing microcystin-producing *Anabaena/Dolichospermum* in the rumen contents.

## 4 Conclusions

Toxicology, histopathology and molecular studies presented here demonstrate that the deaths of 32 steers over a few days in June 2017 in southeastern Oregon were the result of acute liver poisoning by microcystin-LR produced by a single strain of *Anabaena/Dolichospermum,* which we refer to as *Anabaena* sp. JUN03. This cyanobacterium is closely related to *Anabaena* sp. 90 (Fig. 4), a microcystin producer isolated in Finland (Sivonen et al., 1992), and a member of the recently recognized ADA clade of bloom-forming Nostocales (Driscoll et al., 2018). Microcystin-producing harmful algal blooms (HABs) are well known in the nearby Klamath Basin spanning the Oregon/California border, where Upper Klamath Lake, Copco and Iron Gate Reservoirs, and the interconnecting Klamath River, annually suffer highly toxic *Microcystis* blooms (Bozarth et al., 2010, KBMP, 2018, Moisander et al., 2009, Otten et al., 2015). However, no evidence for the presence of *Microcystis* could be found in Junipers Reservoir water samples, indicating the lethal action of a non-*Microcystis* microcystin producing cyanobacterium previously unrecognized in this region.

The potency of *Anabaena* sp. JUN03 as a producer of the highly toxic microcystin-LR serves notice that *Anabaena/Dolichospermum* should be considered as a source of high levels of microcystins in HABs. Indeed, *Anabaena lemmermannii* has been associated with extremely high levels of microcystins (26,180 μg/L) in Lake Biale, Poland (cited in (Kobos et al., 2013). Further, although previous reports limited occurrences to northern latitudes, such as Poland (Kobos et al., 2013), Scandinavia (Rantala et al., 2006, Rapala et al., 1997, Sivonen et al., 1992) and Canada (Harada et al., 1991, Krishnamurthy et al., 1986), our discovery in the moderate latitudes of southern Oregon (42° N) indicates that microcystin-producing *Anabaena/Dolichospermum* should be considered capable of forming dangerous HABs across temperate regions. Our findings validate the requirements that have been instituted in some jurisdictions for microcystin testing when *Anabaena/Dolichospermum* blooms are present.

## Data statement

A fasta file with the gene sequences for cpcBA, mcy, rbcL and rboB is provided.

A GenBank file is provided for the mcy gene cluster, providing gene annotations.

## Ethics statement

Theo Dreher and the coauthors state that this work submitted here has not been submitted for publication elsewhere. All work was conducted to the ethical standards outlined in the Publishing Ethics Resource Kit associated found on the journal webpage.

## Manuscript contributions

TWD conceived the study, conducted bioinformatic analyses and wrote the paper; LPC conducted laboratory and bioinformatic analyses; RSM conducted bioinformatic analyses; PS collected samples and field data; AS conducted toxin analyses; RJB conducted postmortem analyses; RF collected samples and made field diagnoses. All authors contributed to manuscript editing.

## Declaration of interests

The authors declare that they have no known competing financial interests or personal relationships that could have appeared to influence the work reported in this paper.
