# Supplementary material for: Anabaena/Dolichospermum as the source of lethal microcystin levels responsible for a large cattle toxicosis event
Source: Toxicon X. 2018 Dec 10;1:100003. doi: 10.1016/j.toxcx.2018.100003 (PMC7286090; doi:10.1016/j.toxcx.2018.100003)
Supplement: Multimedia component 3 [file mmc3.docx]

**Supplementary Table S1** Precursor and product ions monitored using EPI can mode for quantitative analysis for microcystins LA, LR, RR and YR by LC-MS/MS.

| **Compound** | **Precursor ion (m/z)** | **Product ion(s) (m/z)** |
| --- | --- | --- |
| MC-LA | 910 [M+H]^+^ | 758, 776 |
| MC-LR | 995 [M+H]^+^ | 599 |
| MC-LR | 498 [M+H]^2+^ | 861, 862 |
| MC-RR | 520 [M+H]^2+^ | 620 |
| MC-YR | 523 [M+H]^2+^ | 911, 912 |

**Supplementary Table S2** Serum biochemical analysis conducted on steer sampled just prior to death from acute poisoning, 19 June, 2017. Abnormal values are indicated in red.

| Analyte | Concentration in steer serum | Normal concentration range |
| --- | --- | --- |
| Complete blood count | | |
| White blood cells, total | 26,290 /µL | 4,000 – 12,000 /µL |
| Red blood cells | 9.81 x 10e6/µL | 5 – 10 x 10e6/µL |
| Hemoglobin | 11.3 g/dL | 8 – 15 g/dL |
| Hematocrit | 36.5 % | 24 – 46 % |
| PCV | 33 % | 24 – 46 % |
| MCV | 37.2 fL | 40 – 60 fL |
| MCH | 11.5 pg | 11 – 17 pg |
| MCHC | 31.0 d/dL | 30 – 36 g/dL |
| RDW | 22.8 % |  |
| Platelet count | 158 x 1000/µL | 100 – 800 x 1000/µL |
| Platelet comment | clumped |  |
| Plasma protein | 7.7 g/dL | 6.0 – 8.0 g/dL |
| Fibrinogen | 400 mg/dL | 100 – 600 mg/dL |
| Liver Chemistry profile | | |
| BUN, blood urea nitrogen | 20 mg/dL | 2 – 18 mg/dL |
| Total protein | 7.0 g/dL | 6.0 – 8.4 g/dL |
| Albumin | 3.6 g/dL | 3.1 – 4.1 g/dL |
| Bilirubin, total | 2.8 mg/dL | 0.1 – 0.5 mg/dL |
| Creatine kinase | 653 U/L | 47 – 303 U/L |
| Alkaline phosphatase | 1102 U/L | 30 – 190 U/L |
| Gammaglutamyl transferase | 100 U/L | 1 – 31 U/L |
| Aspartate aminotransferase | >5000 U/L | 51 – 114 U/L |
| Sorbitol dehydrogenase | >170 U/L | 0 – 50 U/L |

**Supplementary Table S3** Cyanotoxin sequences used for mapping metagenome reads.

| **Cyanotoxin genes** | **Source organism** | **GeneBank accession number** | **Base pairs** |
| --- | --- | --- | --- |
| *Anabaena* microcystin | *Anabaena* sp. 90 | AJ536156 | 57,817 |
| *Microcystis* microcystin | *Microcystis aeruginosa* PCC 7806 | [AF183408.1](https://www.ncbi.nlm.nih.gov/nucleotide/AF183408?report=genbank&log$=nuclalign&blast_rank=1&RID=BSA7999Y014) | 63,626 |
| *Planktothrix* microcystin | *Planktothrix agardhii* NIVA-CYA 126/8 | [AJ441056.1](https://www.ncbi.nlm.nih.gov/nucleotide/AJ441056?report=genbank&log$=nuclalign&blast_rank=1&RID=BSAB0YVF015) | 55,575 |
| *Anabaena* anatoxin-a | *Anabaena* sp. 37 | JF803645.1 | 29,504 |
| *Oscillatoria* anatoxin-a | *Oscillatoria* sp. PCC 650 | [FJ477836.2](https://www.ncbi.nlm.nih.gov/nucleotide/FJ477836?report=genbank&log$=nuclalign&blast_rank=1&RID=BSAGAKMY015) | 28,910 |
| *Aphanizomenon* cylindrospermopsin | *Aphanizomenon* sp. 10E6 | [GQ385961.1](https://www.ncbi.nlm.nih.gov/nucleotide/GQ385961?report=genbank&log$=nuclalign&blast_rank=1&RID=BSAMMD6U014) | 57,112 |
| *Cylindrospermopsis* cylindrospermopsin | *Cylindrospermopsis raciborskii* AWT205 | EU140798 | 50,355 |
| *Oscillatoria* cylindrospermopsin | *Oscillatoria* sp. PCC 6506 | [FJ418586.4](https://www.ncbi.nlm.nih.gov/nucleotide/FJ418586?report=genbank&log$=nuclalign&blast_rank=1&RID=BSP99AUD014) | 44,646 |
| *Raphidiopsis* cylindrospermopsin | *Raphidiopsis curvata* HB1 | [KJ139745.1](https://www.ncbi.nlm.nih.gov/nucleotide/KJ139745?report=genbank&log$=nuclalign&blast_rank=1&RID=BSPC4KDZ01R) | 42,821 |
| *Nodularia* nodularin | *Nodularia spumigena* strain NSOR10 | AY210783.2 | 54,353 |
| *Nostoc* nodularin | *Nostoc* sp. 73.1 | [JF342711.1](https://www.ncbi.nlm.nih.gov/nucleotide/JF342711?report=genbank&log$=nuclalign&blast_rank=1&RID=BSNZ3JNV014) | 14,288 |
| *Cylindrospermopsis* saxitoxin | Cylindrospermopsis raciborskii T3 | [DQ787200.1](https://www.ncbi.nlm.nih.gov/nucleotide/DQ787200?report=genbank&log$=nuclalign&blast_rank=1&RID=BSNTKUGU014) | 37,606 |

| **Organism** | **Read Depth** | **St. Dev. of Depth** | **Bases Covered** | **Breadth of Genome Coverage** | **NCBI Genbank Assembly Accession** |
| --- | --- | --- | --- | --- | --- |
| ***Anabaena* sp 90** | 962 | 745 | 3,476,477 | 0.80 | GCA_000312705.1 |
| ***Anabaena*** **sp** **AL09** | 692 | 251 | 3,955,151 | 0.85 | GCA_001672255.1 |
| **Anabaena** **sp** **AL93** | 517 | 640 | 3,687,083 | 0.65 | GCA_001672085.1 |
| ***Aphanizomenon flos aquae*** **WA102** | 302 | 518 | 3,204,391 | 0.54 | GCA_001672105.1 |
| ***Dolichospermum circinale*** **AWQC310F** | 218 | 445 | 2,184,393 | 0.50 | GCA_000426925.1 |
| ***Dolichospermum circinale*** **AWQC131C** | 213 | 439 | 2,133,773 | 0.48 | GCA_000426905.1 |
| ***Anabaena cylindrica*** **PCC** **7122** | 42.4 | 228 | 692,365 | 0.11 | GCA_000317695.1 |
| ***Nostoc azollae*** **0708** | 22.4 | 168 | 359,662 | 0.067 | GCA_000196515.1 |
| ***Nodularia spumigena*** **CCY9414** | 21.6 | 181 | 245,230 | 0.045 | GCA_000340565.3 |
| ***Cylindrospermum stagnale*** **PCC** **7417** | 12.7 | 169 | 172,917 | 0.025 | GCA_000317535.1 |
| ***Nostoc*** **sp** **PCC** **7524** | 7.07 | 128 | 79,167 | 0.012 | GCA_000316645.1 |
| ***Nostoc*** **sp** **PCC** **7107** | 6.82 | 121 | 67,536 | 0.011 | GCA_000316625.1 |
| ***Synechococcus*** ***elongatus*** **PCC** **7942** | 6.45 | 164 | 7,357 | 0.003 | GCA_000012525.1 |
| ***Synechococcus elongatus* PCC** **6301** | 6.45 | 164 | 7,357 | 0.003 | GCA_000010065.1 |
| ***Nostoc punctiforme*** **PCC** **73102** | 5.63 | 111 | 91,343 | 0.011 | GCA_000020025.1 |
| ***Calothrix sp*** **PCC** **6303** | 4.12 | 107 | 36,025 | 0.005 | GCA_000317435.1 |
| ***M****i****crocystis aeruginosa*** **NIES** **2481** | 3.90 | 133 | 10,165 | 0.002 | GCA_001704955.2 |
| ***Microcystis aeruginosa*** **NIES** **2549** | 3.90 | 133 | 9,291 | 0.002 | GCA_000981785.2 |
| ***Cylindrospermopsis raciborskii*** **CS** **50**5 | 3.76 | 43.0 | 4,035 | 0.016 | GCA_001676585.1 |
| ***Microcystis aeruginosa*** **NIES** **843** | 2.92 | 116 | 13,192 | 0.002 | GCA_000010625.1 |
| ***Raphidiosis brookii*** **D9** | 2.61 | 85.9 | 4,225 | 0.008 | GCA_001900715.1 |
| ***Richelia intracellularis*** **HH01** | 0.0169 | 0.563 | 0 | 0 | GCA_000350105.1 |

**Supplementary Table S4** Mapping of JUN03 metagenome sequencing reads against reference cyanobacterial genomes with Bowtie2, showing average and standard deviation of read depths, number of bases in the reference genome covered, and breadth of coverage (proportion of reference genome).

**Supplementary Table S5** PCR primers used in these studies.

| **Primer** | **Gene target** | **Sequence** | **Exp. Product Size (bp)** | **Annealing temp. (ºC)** | **Source** |
| --- | --- | --- | --- | --- | --- |
| Ana 573F | 16S rDNA | AGTGGAAACTACAAAGCTAGAGTT | 216 | 55 | Doblin et al., 2007 |
| Ana 780R |  | CTTGGGTCGATACGAGCT |  |  |  |
| **McyBir-F** | *mcyB* & *mcyC* | ACCGTGATTGGCACATCATT | 381 | 58 | This paper |
| **McyBir-R** |  | TTCGCAACATTGAGGGAAC |  |  |  |
