# Supplementary material for: Anabaena/Dolichospermum as the source of lethal microcystin levels responsible for a large cattle toxicosis event
Source: Toxicon X. 2018 Dec 10;1:100003. doi: 10.1016/j.toxcx.2018.100003 (PMC7286090; doi:10.1016/j.toxcx.2018.100003)

Suppl. Fig. S1 *Anabaena/Dolichospermum* present in scum-containing water sample JUN01 from Junipers Reservoir. **A, B.** Akinetes and heterocysts embedded in collapsed tangles of vegetative cells. 400x. A short straight filament at right of image A is suggestive of *Aphanizomenon flos-aquae*.

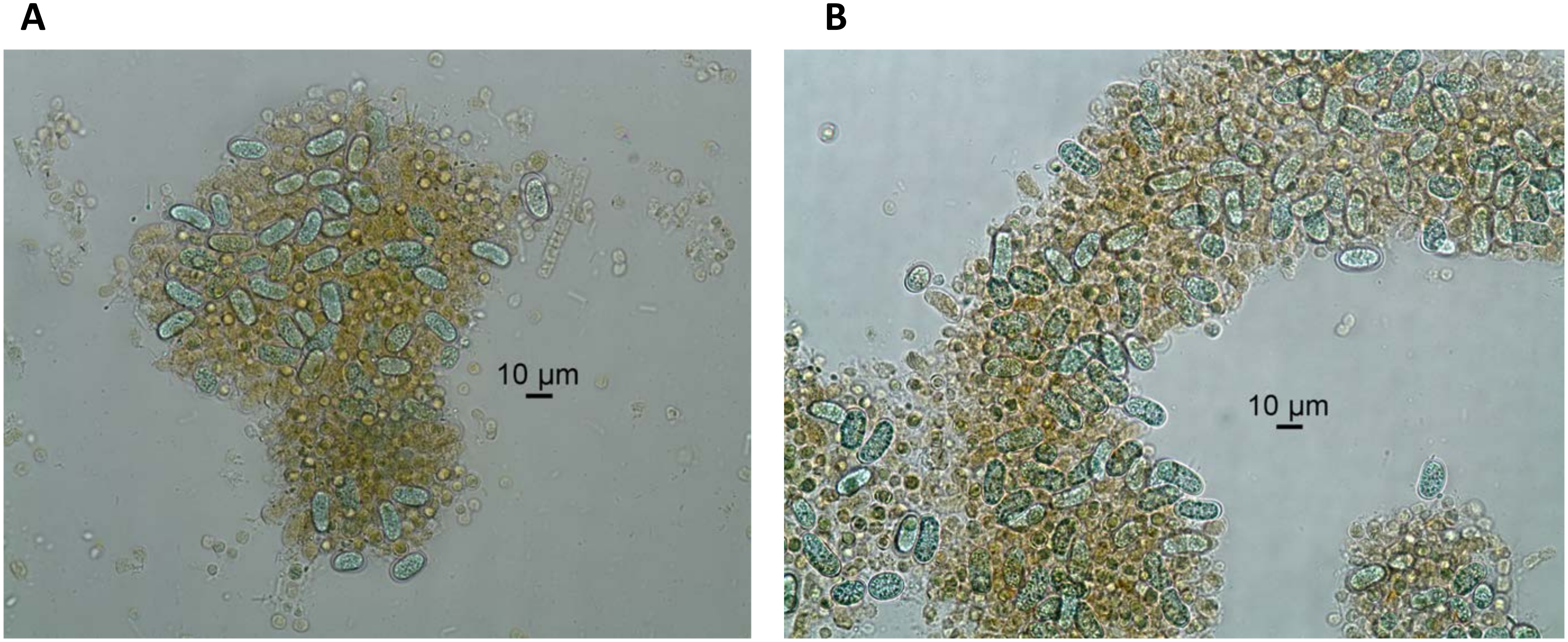

Suppl. Fig. S2 PCR products made from rumen, JUN01 and JUN03 samples using primers (Table S4) specific for *mcyB* + *mcyC* and for 16S rDNA genes, separated by electrophoresis on a 1.5% agarose Tris-acetate gel. Marker DNA sizes are indicated at right in bp. Each band of the *mcy* doublet produced from JUN03 DNA was excised and sequenced, showing the lower band to be derived from *mcyB* and the upper band from *mcyC*. A 72 bp insertion explains the product size difference between the two bands. Two PCR amplifications between primers base-pairing inside and outside the insertion were used to verify the presence of the *mcyC* insertion (not shown).

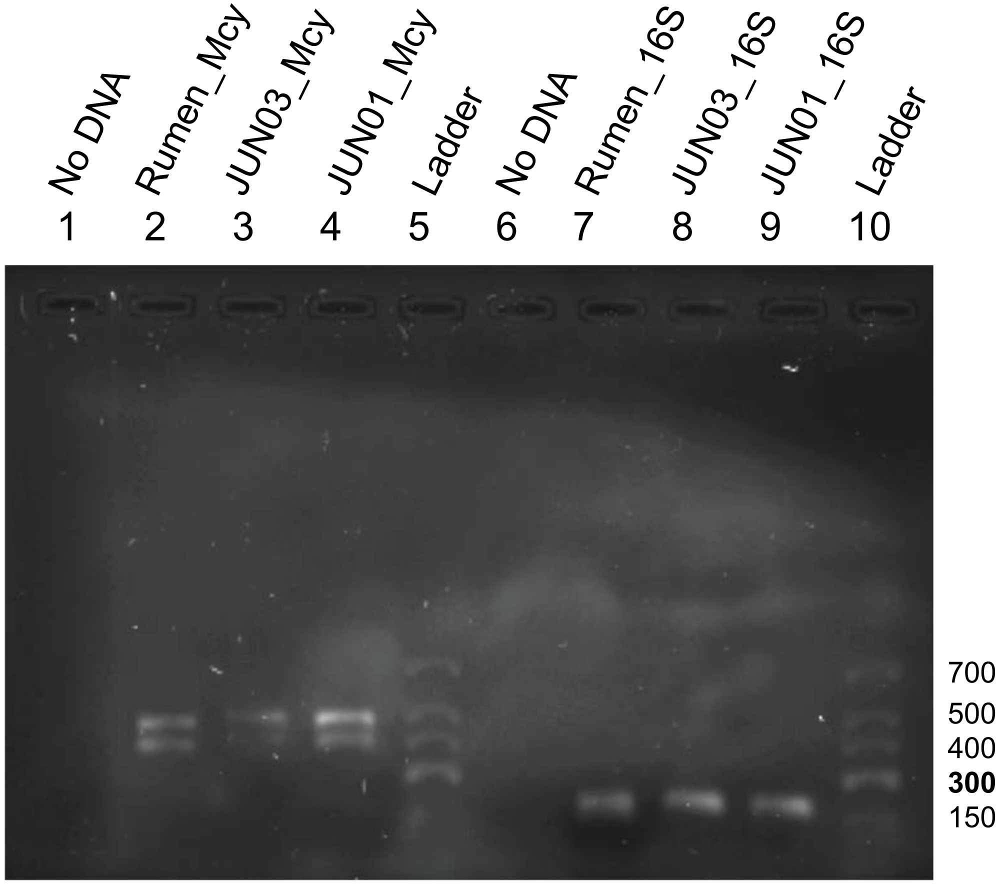

Suppl. Fig. S3 The 55-kbp JUN01/JUN03 *mcy* gene cluster showing PKS (gray), NRPS (blue) and tailoring domains (green) predicted by antiSMASH v4.1.0. The 72 bp insertion relative to the *mcyC* gene of *Anabaena* sp. 90 is indicated. PKS domains: ACP, acylcarrier protein; AT, acyltransferase; DH, dehydratase; KR, ketoreductase; KS, ketosynthase AMP. NRPS domains: , AMP-binding adeylation domain, C, condensation domain; PCP, peptide carrier protein (thiolation). Tailoring domains: AminoTrans, aminotransferase; E, epimerization; cMT, C-methylation; oMT, O-methylation.

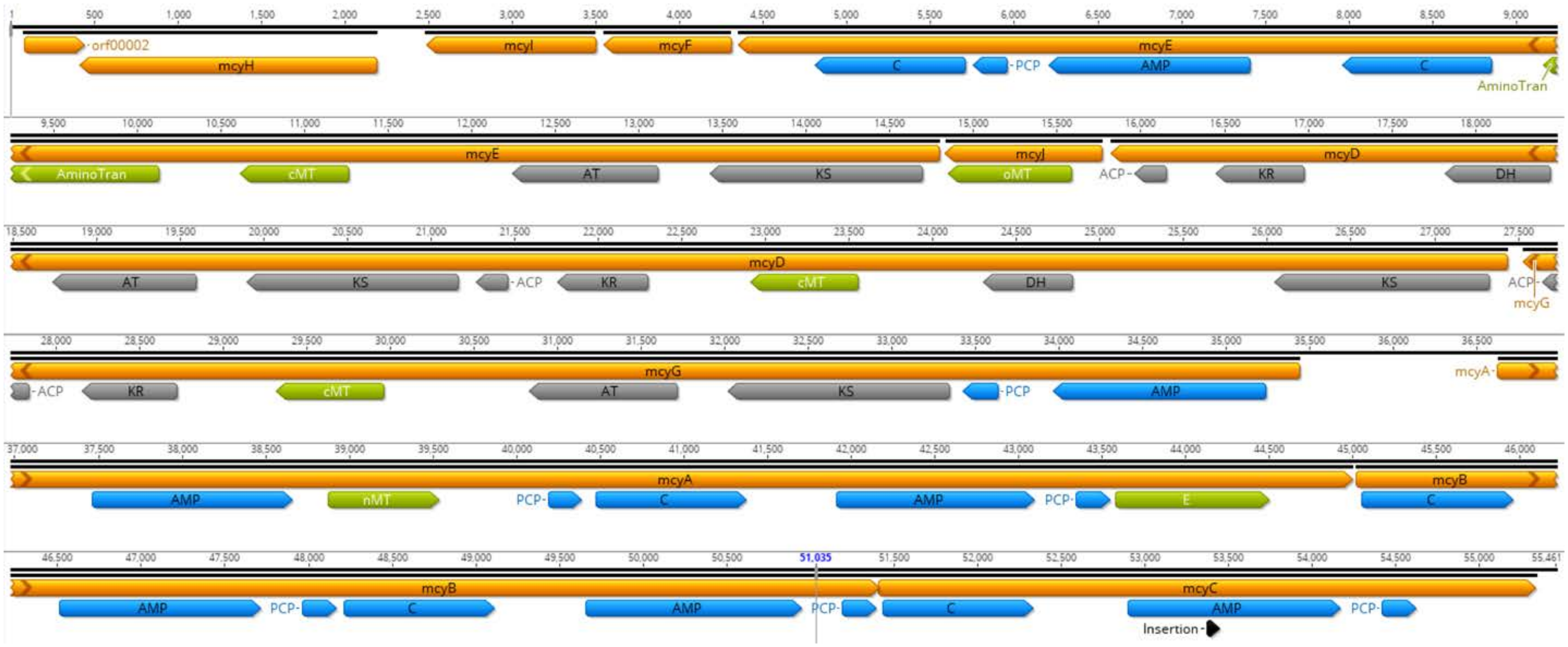

Supplement: Multimedia component 4 [file mmc4.pdf]
